# Real world challenges and barriers for positive airway therapy use in acute ischemic stroke patients

**DOI:** 10.1007/s11325-024-03161-7

**Published:** 2024-09-16

**Authors:** Maeve Pascoe, Madeleine M Grigg-Damberger, Harneet Walia, Noah Andrews, Lu Wang, James Bena, Irene Katzan, Ken Uchino, Nancy Foldvary-Schaefer

**Affiliations:** 1 Department of Neurology, Sleep Disorders Center, Cleveland Clinic Neurological Institute, 9500 Euclid Avenue, S73, Cleveland, OH 44195 USA; 2 Department of Neurology, University of New Mexico, Albuquerque, NM USA; 3 Quantitative Health Sciences, Cleveland Clinic, Cleveland, OH USA; 4 Department of Neurology, Cerebrovascular Center, Cleveland Clinic, Cleveland, OH USA; 5 Baptist Hospital, Miami, FL USA

**Keywords:** Stroke, Sleep disordered breathing, Portable monitoring, PAP acceptance

## Abstract

**Purpose:**

Untreated obstructive sleep apnea (OSA) in patients with acute ischemic stroke (AIS) is associated with increased morbidity and mortality. However, diagnosing and treating OSA in AIS is challenging. We aimed to determine the feasibility of portable monitoring (PM) for diagnosis and positive airway pressure therapy for treatment of OSA in an inpatient stroke population.

**Methods:**

We recruited inpatients with AIS from Cleveland Clinic. Those who consented underwent PM; participants with a respiratory event index (REI) ≥ 10 were offered auto-titrating positive airway pressure therapy (APAP). Ease-of-use questionnaires were completed. We summarized categorical variables using n(%) and continuous variables using mean ± SD or median [IQR].

**Results:**

27 participants (age 59.8 ± 11.8, 51.9% female, 51.9% Black, BMI 33.4 ± 8.5) enrolled. The study ended early due to Medicare contracting that forced most patients to complete stroke rehabilitation outside the Cleveland Clinic health system. 59.3% had large vessel occlusions and 53.8% had moderate/severe disability (Modified Rankin score ≥ 2). PM was attempted in 21 participants, successful in 18. Nurses and patients rated the PM device as highly easy to use. 13 of 18 (72%) patients who had an REI ≥ 10 consented to APAP titration, but only eight (61.5%) of those 13 used APAP for more than one night, and only five (27.8%) used APAP up to 90 days with data captured for only one participant. Five required troubleshooting at titration, and only one had adherent APAP usage by objective assessment after discharge.

**Conclusions:**

This study demonstrates the real-world challenges of assessing and treating OSA in an AIS population, highlighting the necessity for further research into timely and feasible screening and treatment.

## Introduction

Approximately 50–70% of patients with acute ischemic stroke (AIS) or transient ischemic attack (TIA) have obstructive sleep apnea (OSA) [1]. Large prospective population-based studies have established that OSA is an independent risk factor for incident stroke [2–6]. In previous studies, an apnea-hypopnea index (AHI) ≥5 events/hour of sleep was shown to independently increase the risk for stroke or death by 1.9 times after adjusting for confounders [4]. Additionally, men with an obstructive AHI ≥20 had a 2.9-fold higher risk for incident stroke compared to those with AHI < 5; in women, increased risk for stroke was observed in those with an obstructive AHI > 25 [3]. 

Stroke patients who have OSA have worse cognitive functioning and functional capacity, reduced mobility, greater impairment in activities of daily living (ADLs), and longer stays in inpatient rehabilitation than those without OSA [7–10]. OSA patients recovering from stroke have poorer psychomotor skills, longer hospital recovery time, more depressive symptoms, and increased all-cause mortality compared to stroke patients without OSA [2, 10]– [13]. 

The detrimental effects of OSA on stroke outcomes could potentially be mitigated by earlier diagnosis and effective treatment. Two randomized controlled trials showed that the greatest improvements in neurological recovery were observed among stroke patients who started positive airway pressure (PAP) within 48 h of stroke compared to those who started PAP later [14, 15]. However, numerous studies have found treating OSA in AIS challenging in all stages of the process from recruitment to follow-up [9, 10, 16]– [18]. 

Given the need for assessment and treatment in this population, we designed this prospective study to determine the feasibility, ease, and efficacy of portable cardiorespiratory monitoring (PM) for diagnosis and auto-adjusting positive airway pressure (APAP) treatment in patients admitted to an inpatient stroke unit in a quaternary care center. In the process, we observed barriers to care which we found cautionary but instructive.

## Methods

### Participants and recruitment

Adult patients with AIS supported by clinical determination and brain magnetic resonance imaging (MRI) admitted to the inpatient stroke service at Cleveland Clinic were invited to participate. Potential participants were identified using inpatient census lists. Exclusion criteria included severe insomnia, circadian rhythm disorders, unstable medical conditions, major psychiatric conditions (aside from depression) that would interfere with study participation, suicide attempt within 3 months, severe chronic obstructive pulmonary disease/severe restrictive lung disease, daily narcotic use, previous PAP therapy use within 3 months of stroke, and inability to provide consent/obtain consent from a healthcare power of attorney. The study was approved by the Cleveland Clinic Institutional Review Board.

### Study design

Eligible participants provided informed consent prior to completion of study procedures. Thereafter, baseline characteristics were collected and PM was performed. Following PM, both patients and nursing staff completed PM ease-of-use questionnaires. Participants with a respiratory event index (REI) ≥ 10 were considered to have OSA and were offered APAP therapy. Those who consented underwent APAP titration and treatment and completed questionnaires about PAP ease of use. Follow-up was planned following discharge in those who agreed to use PAP therapy.

### Study procedures

Baseline characteristics included: (1) demographics (age, gender, race), medical history, and medication history; (2) physical examination and vital signs with body mass index [BMI]; (3) patient-reported and provider-reported assessments of stroke and sleep characteristics; and (4) stroke characteristics including MRI findings and presence of wake-up stroke.

Patient-reported outcomes were completed. The Patient Health Questionnaire-9 (PHQ-9) is a depression scale based on the diagnostic criteria for major depressive disorder in the DSM-IV; a score of ≥ 10 is indicative of moderate-to-severe depressive symptoms [19]. The Epworth Sleepiness Scale (ESS) is a questionnaire that measures subjective daytime sleep propensity by asking participants how likely they are to fall asleep in different situations; a score of ≥ 10 is indicative of excessive daytime sleepiness [20]. The Fatigue Severity Scale (FSS) is a validated method for assessing fatigue; a mean score of ≥ 36 is suggestive of significant fatigue [21]. The Functional Outcomes of Sleep Questionnaire (FOSQ) measures the impact of sleep on functional status with daily activities; a score of ≤ 18 indicates an abnormal impact of sleepiness on ADLs [22]. 

MRI results were used to categorize the stroke (dominant/non-dominant hemisphere stroke, cortical/subcortical/brainstem/cerebellum localization). Healthcare providers noted the date of the last stroke event, stroke type and mechanism (including suspected wake-up stroke). Participants completed stroke assessments including the Stroke Impact Scale-16 (SIS-16), designed to measure ADLs and shown to have better discrimination between levels of disability and fewer ceiling effects than the provider-reported Barthel Index [23]. The modified Rankin Scale measures function in ADLs in patients who have had a stroke/TIA with higher item scores indicating a greater degree of disability; a score of > 2 indicates moderate-to-severe disability [24]. The Barthel Index assesses the level of autonomy in ADLs; a score < 35 corresponds with high risk for dependency on others for ADLs, 35–70 medium risk, and > 70 low risk [25]. The NIH Stroke Scale (NIHSS) is a widely used scale quantitatively assessing performance on the standard neurological exam; a maximum of 42 points is possible, with a score of 1–4 indicating minor stroke, 5–15 moderate, 16–20 moderate-severe, and 21–42 severe stroke [26]. 

PM was performed using the CleveMed SleepView™, a Type 3 portable sleep PSG system that records snoring, thermal and nasal pressure airflow, respiratory effort, pulse oximetry, heart rate, and body position. Presence of OSA was defined as a REI ≥ 10 events/ hour. After PM, both participants and nursing staff completed the PM Ease of Use questionnaire using a Likert scale (1 = strongly disagree to 5 = strongly agree). Participants with OSA on PM underwent PAP education and mask fitting followed by an auto-titration using a ResMed APAP device. Participants without OSA or who refused PAP therapy exited the study.

PAP therapy for OSA participants was initiated in the form of APAP. APAP was titrated for at least one night, and the titration was considered acceptable if: (1) the recording period averaged ≥ 4 h; (2) REI was less than that recorded on the diagnostic PM; and (3) excessive leak (≥ 2 times the 95th percentile leak at the 95th percentile pressure for the interface) was absent. If the initial titration was unacceptable, the titration was repeated for an additional night. From there, either acceptable results were obtained or the participant exited the study. A technologist provided daily education to participants, family members and nurses to ensure optimal hours of usage and provide PAP troubleshooting including mask adjustments and replacement and desensitization. The number of troubleshooting interventions was recorded over the period of study participation.

If discharge was planned before acceptable APAP titration, participants were provided an APAP machine with an extra SD card and a self-addressed envelope to perform the study at home and return the SD card by mail. Telephone contact was made in these cases to ensure the APAP study had been completed and the SD card returned, with a second contact made to review the results of the download. After hospital discharge, those who accepted APAP therapy were contacted by phone to assess PAP adherence and complete the APAP Ease of Use questionnaire. Adherence was defined based on the Centers for Medicare and Medicaid Services (CMS) adherence criteria (≥ 4 h of use on ≥ 70% of nights).

### Statistical analysis

Categorical variables were summarized with n(%) while continuous variables were summarized using mean ± SD or median [IQR], as appropriate. Study data was collected and managed using REDCap, a secure web-based software platform designed to support data capture for research studies [27, 28]. Statistical analysis was performed using Microsoft Excel [version 15.0.5]. Microsoft; 2013.

## Results

**Fig. 1:**
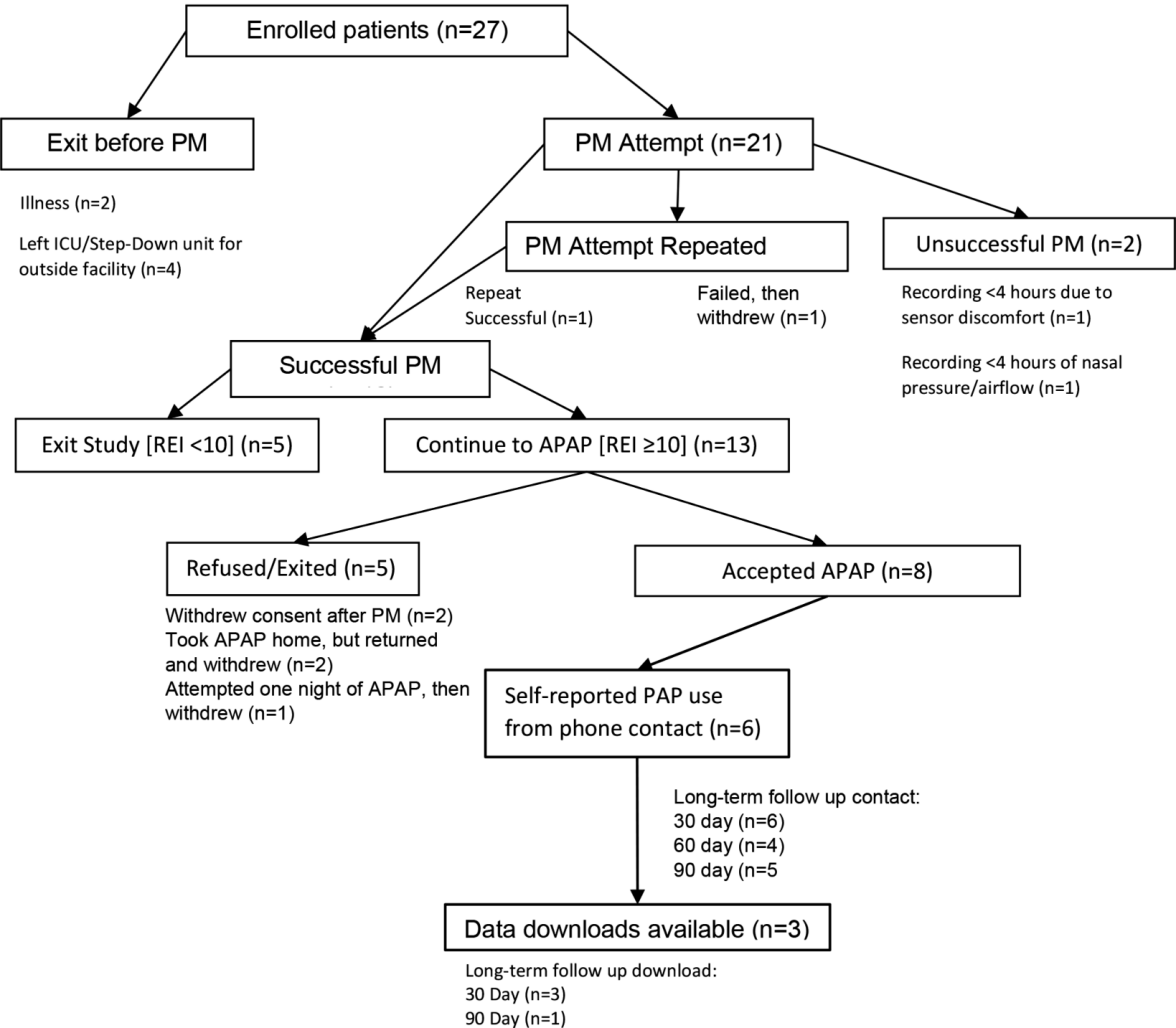
Study Flowchart - Tracking Enrollment to PM to APAP

A flowchart of participant involvement is shown in Fig. 1. Sample characteristics are shown in Table 1. A total of 27 patients were enrolled (age 59.8 ± 11.8, 51.9% female, 51.9% Black). Post-hoc analysis revealed that no patient had engaged with sleep medicine prior to enrollment and none had sleep apnea in their medical record problem lists, though one patient had a mention of “unspecified sleep apnea” in their general office visit history. Mean time from stroke to first study visit was 4.1 ± 2.7 days. Most participants had hypertension (89%) and 55.6% of strokes were cortical. The majority had stroke deficits that could interfere with PAP use including upper extremity paresis (70.4%) and facial paresis (44.4%).

**Table 1:** Samplecharacteristicss (*n* = 27)

Factor	Statistics
*Demographics*	
Age, yr	59.8 ± 11.8
Gender, % female	14 (51.9)
Handedness, right	25 (93.0)
*Race / Ethnicity*	
Black	14 (51.9)
Caucasian	13 (48.1)
Body Mass Index, kg/m^2^	33.4 ± 8.5
*Co-morbidities*	
Hypertension	24 (89.0)
Coronary artery disease	5 (18.5)
Hyperlipidemia	13 (48.1)
Atrial Fibrillation	6 (22.2)
Diabetes Mellitus	13 (48.1)
*Stroke Characteristics*	
Time from neuro step-down unit admittance to signed consent median, days	4.1 ± 2.7
Localization	
Hemisphere of stroke, Left	15 (55.6)
Dominant hemisphere	11 (40.7)
Cortical	15 (55.6)
Classification	
Large-artery atherosclerosis (embolus/thrombosis)	16 (59.3)
Cardioembolism	3 (11.1)
Small-vessel occlusion (lacunar)	6 (22.2)
Wake-up stroke	9 (33.3)
*Pertinent Exam Findings*	
Aphasia	4 (14.8)
Dysarthria	11 (40.7)
Upper extremity weakness	19 (70.4)
Upper extremity sensory deficit	4 (14.8)
Facial weakness	12 (44.4)
Facial sensory deficit	4 (14.8)

Statistics presented as Mean ± SD, Median [P25, P75] or N (%)

Stroke outcomes and sleep characteristics are detailed in Table 2. The median Modified Rankin was 2 [IQR 2,2]; More than a third (34.6%) of participants had a score of 2 indicating slight disability, and 19.2% had scores > 2 indicating moderate-to-severe disability. The median NIHSS total score was 4, indicating mild stroke, but strokes across the cohort ranged from mild to moderate [1.5-8.0]. The median SIS-16 was 59.0 [42.0, 68.0], which is better than previously published means for stroke [29], and the median Barthel Index was 100 [80, 100], indicating a high level of functioning. Baseline sleep characteristics were notable for 50.0% having elevated ESS scores indicating abnormal daytime sleep propensity, 62.5% having excessive fatigue and 88% having low FOSQ scores indicative of quality of life impairments related to sleep. The majority (92%) had at least mild depression on the PHQ-9.

**Table 2:** Post-strokee and sleepcharacteristicss

	Total (*N* = 27)	
Factor	*n*	Statistics
*Post-Stroke characteristics*		
Modified Rankin Scale score	26	2 [2,2]
Modified Rankin Scale score ≥ 2	26	14 (53.8)
Modified Rankin Scale score > 2	26	5 (19.2)
NIHSS Total Score	27	4 [1.5, 8.0]
SIS-16	25	59 [42, 68]
Barthel Index	25	100 [80, 100]
*Sleep*,* mood*,* and functioning characteristics*		
Epworth Sleepiness Scale (ESS)	24	9.4 ± 5.2
ESS ≥ 10	24	12 (50.0)
Fatigue Severity Scale (FSS)	24	39.5 ± 15.6
FSS ≥ 36	24	15 (62.5)
Functional Outcomes of Sleep Questionnaire (FOSQ)	25	14.2 [10.8,17.6]
FOSQ *≤* 18	25	22 (88.0)
Patient Health Questionnaire-9 (PHQ-9)	25	17.8 ± 7.0
PHQ-9 ≥ 10	25	23 (92.0)

Statistics presented as Mean ± SD, Median [P25, P75] or N (%)

PM was attempted in 21 participants; the 6 patients for whom PM was not attempted due to disease severity/clinical instability (*n* = 2) or were transferred out of the step-down unit or discharged (*n* = 4). Of the 21 that attempted PM, 18 were successful (85.7%) One participant’s data was incomplete due to data corruption. Two patients’ data did not meet study criteria to continue to APAP titration (*≤* 4 h of recording time): one recording was terminated by the patient due to sensor discomfort and the other was missing an acceptable nasal pressure/airflow signal. Table 3 reflects the 18 patients that had PM data. The median REI was 18.3 [10.6, 24.5]; 13 (68.4%) met criteria for OSA based on an REI ≥ 10. Two participants had minimal Cheyne-Stokes breathing but neither had significant central apnea. Participants and nurses rated the PM device as highly easy to use (4.8/5.0 ease overall), and most (69.6%) would recommend the device to others.

**Table 3:** Portable monitoring results

	Total (*N* = 18)	
Factor		Statistics
Total recording time, min		489.6[449.0,533.1]
Respiratory Event Index (REI)		18.3[10.6,24.5]
REI ≥ 10		14 (77.8)
REI Supine		17.3[7.4,33.1]
REI Off-supine		16.1[5.8,29.1]
Apnea Index		0.81[0.00,5.1]
Hypopnea Index		11.4[7.2,19.4]
Central Apnea Index		0.70[0.00,2.4]
Baseline SpO2, %		97.0[96.0,97.0]
Mean SpO2, %		96.4[96.0,96.7]
Minimum Sp02, %		71.25[63.73, 77.65]
Recording Time with Sp02 < 90%, %		0.55[0.01,1.8]
Cheyne Stokes Breathing Pattern		2(11.1)
Snoring		13(72.2)

Statistics presented as Mean ± SD, Median [P25, P75] or N (%)

Of the 13 participants with a REI ≥ 10, all agreed to attempt APAP titration. Of these, 2 withdrew consent after completing PM, 2 were non-compliant with titration, and 1 returned to the neurologic intensive care unit due to subsequent stroke, leaving 8 (61.5%) participants that accepted APAP treatment for discharge. Of the 8, troubleshooting was needed during hospitalization for five (62.5%) participants for poor mask fit (3/5), mask leak (3/5), and PAP re-education/reinforcement (4/5), while troubleshooting continued after discharge for two of the five for mask fit/humidity problems. Of the five that had troubleshooting, four participants received new masks and a change of their humidification settings. However, one of the five that underwent troubleshooting could not successfully initiate PAP therapy and ended their participation in the study without providing any PAP adherence data.

After discharge, five patients reported their daily average PAP use at one week, six patients at 30 days, four at 60 days, and five patients after 90 days; data was only available for three at 30 days and one at 90 days. Data from the APAP device downloads showed that at 30 days, all 3 patients’ AHI values had normalized, but mask leak was greater than the maximum acceptable in one patient. Additionally, at 90 days, the one patient for whom data was still being collected continued to have a normal AHI and acceptable mask leak, but with suboptimal average PAP use of 3.1 h per day. Three of the eight who initiated APAP after discharge completed the APAP ease-of-use questionnaire. All reported the device was easy to use, but one reported problems from their stroke that interfered with APAP use. All three said that the mask did not disturb their sleep, but problems with mask fit, leak, pressure, and nasal dryness were reported.

Follow-up was planned to assess stroke and sleep characteristic change, but this proved infeasible due to changes in Medicare contracting that necessitated the use of rehabilitation facilities outside the healthcare system. The study team lost access to most participants during rehabilitation, leading to early study termination.

## Discussion

This feasibility study performed at a quaternary center confirmed that OSA is highly prevalent in patients with AIS and the diagnosis can be confirmed with easy-to-use in-hospital PM. In-hospital PM was well accepted by participants and nurses. While our sample is small, it was diverse with 52% of participants identifying as Black. We were unable to explore sleep health disparities given the early study termination. Also, 100% (13/13) of participants with OSA agreed to in-hospital APAP titration, but only 61.5% (8/13) accepted APAP. Encouraging participants to accept APAP following hospital discharge was most challenging. Last-minute rejections of APAP therapy often occurred before discharge even after successful APAP titration due to persistent difficulties after troubleshooting (5/13, 38.5%). At follow-up, the few participants who agreed to use APAP typically said therapy was easy to use but problems from their stroke interfered with use. Despite troubleshooting, problems with mask fit, leak, and nasal dryness persisted. At the 90-day follow-up, only two of 13 (15.4%) participants with OSA continued to use PAP therapy. Only one of these two patients could be confirmed to use PAP therapy with download data.

We are not the first to encounter dismal acceptance of PAP therapy in patients with AIS [30]. One prospective study of 152 patients with AIS prescribed PAP in 51% but only 15% used it long-term [30]. Anticipating this, we attempted to improve APAP acceptance by providing repeated PAP education, mask desensitization, mask fitting and troubleshooting. However, acceptance remained poor.

What are the barriers to PAP use reported by patients with AIS? Patients with stroke often have poor fit and tolerance of PAP interfaces especially those with facial weakness [16]. Mask placement can be challenging for patients with upper extremity weakness even with preserved sensation. Caregivers can assist in placement of the mask at sleep onset, but awakenings occur that lead to mask removal and patients may have difficulty replacing the mask without assistance. Previous literature suggests that stroke patients must understand their OSA diagnosis and the risks and benefits of treatment and have ample support to attain PAP adherence in order to reduce the risk of stroke recurrence [31]. The accelerated timeline for this study, requiring participants to go through these steps within days of PM, coupled with AIS symptom burden in our cohort, presented too great an obstacle for PAP acceptance.

Stroke patients with OSA as a group do not report lower levels of sleep quality or higher levels of sleepiness or fatigue than stroke patients without OSA [10, 18]. Patients newly diagnosed with stroke can feel overwhelmed by stroke risk factors they are encouraged to work on minimizing. Coping with neurological deficits and the need to optimize blood pressure, body weight, lipid profile and exercise, sleep apnea looms less important.

Should we continue to argue for PAP therapy in patients with moderate/severe OSA following stroke or TIA? Nonrandomized data from the literature suggest significant benefits from PAP therapy in this population. Prior studies have shown that PAP adherence in those with moderate-severe OSA (AHI > 15) after AIS reduces odds for recurrent stroke to 0.13 and is significantly associated with greater survival time [32]. Additionally, a 2021 retrospective cohort study involving 5,757 Medicare beneficiaries aged ≥ 65 years with newly diagnosed OSA showed a 2% reduction in stroke risk with each month of PAP adherence [33]. Furthermore, a study promoting an intensive PAP adherence program in 62 inpatient stroke rehabilitation patients found those who were adherent experienced greater improvements in the cognitive component of the Functional Independence Measure and the NIHSS [34]. While the NIHSS is limited in its ability to show improvements in rehabilitation in all stroke types [35], these findings support continued efforts to assist stroke patients with PAP adherence. As such, it is worthwhile for stroke neurologists, sleep specialists, and primary care physicians to partner together to advocate for early PAP treatment in AIS patients with OSA.

When is the optimal time to start PAP therapy following AIS? Two randomized controlled trials found the greatest improvements in neurological recovery in patients who started PAP within 48 h of stroke onset [14, 15, 36]. A reversed Robin Hood syndrome with a paradoxical decrease in blood flow during episodes of hypercapnia in cerebral blood vessels supplying ischemic areas of the brain and increased blood flow velocity in non-affected vessels was the hypothesized mechanism [37–39]. However, as is seen in this investigation, due to logistic difficulties and post-stroke physical challenges, onset within 48 h may not be feasible.

Recent studies are exploring education techniques to encourage PAP adherence following AIS including motivational interviewing and mobile health messaging [40–42]. Effective treatment of OSA in AIS may be enhanced by OSA-targeted phenotyping therapies [43, 44]. Patients whose OSA is related to low arousal threshold might be treated by a hypnotic; [45] abnormal chemoreflex responses to varying CO2 levels by supplementary oxygen; [46] and low activation of upper airway dilator muscles by hypoglossal stimulation or oropharyngeal exercises [47, 48]. 

Given the heavy burden of morbidity and mortality of OSA on stroke, we need to learn from thwarted studies such as this and explore innovative stroke prevention strategies to improve stroke-free survival [36]. Sleep specialists and organizations need to raise awareness among primary care and stroke neurology clinicians and the general public regarding the role of OSA in stroke/TIA, and work on feasible timelines for PAP therapy initiation in eligible individuals to promote adherence and prevent stroke recurrence and mortality. These factors all contribute to the difficulty of conducting PM and introducing PAP therapy in the hospital setting. As such, diagnostic testing and treatment initiation may be more successful in the rehabilitation setting where most stroke patients exhibit functional improvements and there is more time for education and therapeutic troubleshooting.

## Data Availability

The datasets generated during and/or analyzed during the current study are available from the corresponding author on reasonable request.

## Abbreviations

AIS: Acute ischemic stroke
APAP: Auto-titrating positive airway pressure therapy
ESS: Epworth Sleepiness Scale
FOSQ: Functional Outcomes of Sleep
FSS: Fatigue Severity Scale
MRI: Magnetic resonance imaging
NIHSS: National Institutes of Health Stroke Scale
OSA: Obstructive sleep apnea
PAP: Positive airway pressure therapy
PHQ-9: Patient Health Questionnaire-9
SIS-16: Stroke Impact Scale 16
PM: Portable monitoring
TIA: Transient ischemic attack
